# A Case of Torsion of Meckel’s Diverticulum

**DOI:** 10.7759/cureus.33850

**Published:** 2023-01-16

**Authors:** Katsudai Shirakabe, Ken Mizokami

**Affiliations:** 1 General Surgery, Tokyo Bay Urayasu Ichikawa Medical Center, Urayasu, JPN

**Keywords:** acute abdomen, necrosis, ischemia, laparoscopic surgery, torsion, meckel's diverticulum

## Abstract

Meckel's diverticulum is the most common congenital abnormality of the gastrointestinal tract. Histologically, it is a true diverticulum comprising all four layers of the intestinal tract. The complications associated with Meckel's diverticulum include bleeding, bowel obstruction, and intussusception. Torsions are an extremely rare complication. The patient was a 15-year-old boy who presented to the emergency department with acute-onset lower abdominal pain and was admitted to the hospital for a follow-up of abdominal pain due to nonspecific imaging findings. The symptoms of the patient worsened 12 hours after admission, and he underwent emergency laparoscopic surgery. A large Meckel's diverticulum with torsion and necrosis was observed 30 cm proximal to the ileocecal valve. The diverticulum was twisted around the base of the neck. Subsequently, wedge resection of the small intestine, including the diverticulum, was performed. Stem torsion is a rare complication of Meckel's diverticulum. As definitive preoperative diagnosis was difficult to obtain through imaging studies, early laparoscopic surgery was considered effective.

## Introduction

Meckel's diverticulum is a remnant of the embryologic vitelline duct that connects the fetal gut with the yolk sac, and normally involutes between the fifth and seventh weeks of gestation. Meckel's diverticulum is the most common congenital abnormality of the gastrointestinal tract, occurring in 2% of the population [1]. The lifetime risk of complications from a Meckel's diverticulum is 4%, with the risk decreasing with age [2]. Meckel’s diverticulum can be diagnosed using a technetium scan. Technetium-99m pertechnetate is injected intravenously and accumulates in the gastric mucosa over time [3]. Though it is useful in the diagnosis of bleeding complications, in patients with non-bleeding complications, diagnosis by imaging studies is difficult. Hence, diagnostic laparoscopic surgery is more effective. We report a novel case of Meckel's diverticulum torsion with its pedicle.

## Case presentation

**Figure 1:**
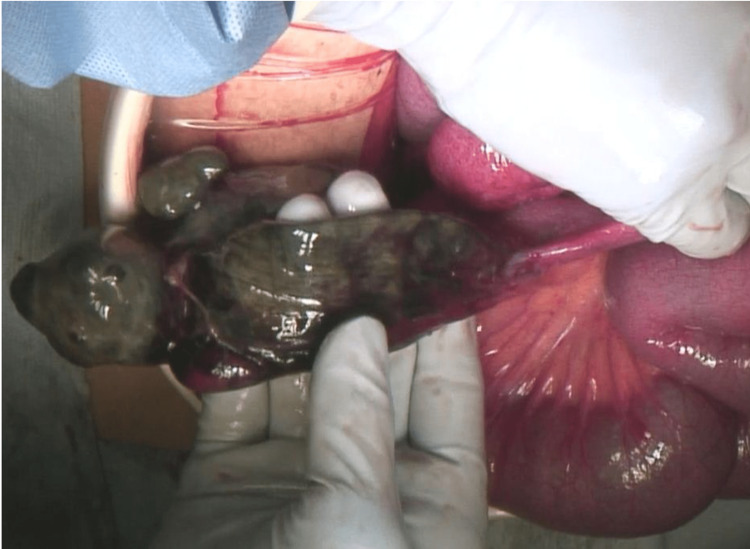
Necrotic Meckel's diverticulum

**Figure 2:**
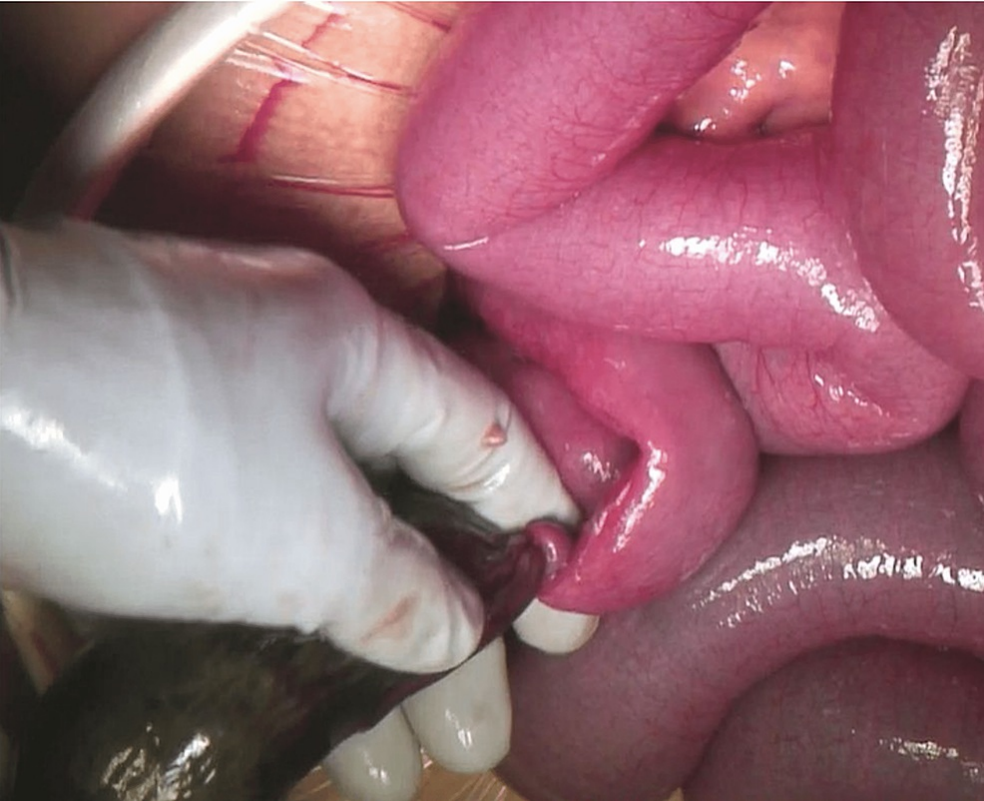
Meckel's diverticulum twisted at the neck

A 15-year-old male came to the emergency department with right lower abdominal pain. The pain was intermittent and there was no constipation or obstipation. The past medical history of the patient was not remarkable. Vital signs were all normal with a temperature of 36.9°C, blood pressure of 153/ 88 mmHg, pulse rate of 90 bpm, and respiratory rate of 14/min. The pH of the blood was 7.332, and the lactate level was 11 mg/dl. On abdominal examination, the abdomen was soft and flat, with intense tenderness in the right lower abdomen, but no recurrent tenderness. Twelve hours after admission, abdominal pain worsened and recurrent tenderness appeared. Hence, laparoscopic surgery was immediately performed to obtain the diagnosis. The laparoscopic surgery revealed a small amount of purulent ascites in the abdominal cavity. Additionally, a necrotic Meckel's diverticulum, approximately 30 cm from the ileum (Figure 1), which was twisted at the neck (Figure 2) was observed. Hence, an additional median incision was made, and approximately 15 cm of twisted and necrotic Meckel's diverticulum was excised. The postoperative clinical course of the patient was uneventful, and he was discharged on the 10th postoperative day.

## Discussion

**Figure 3:**
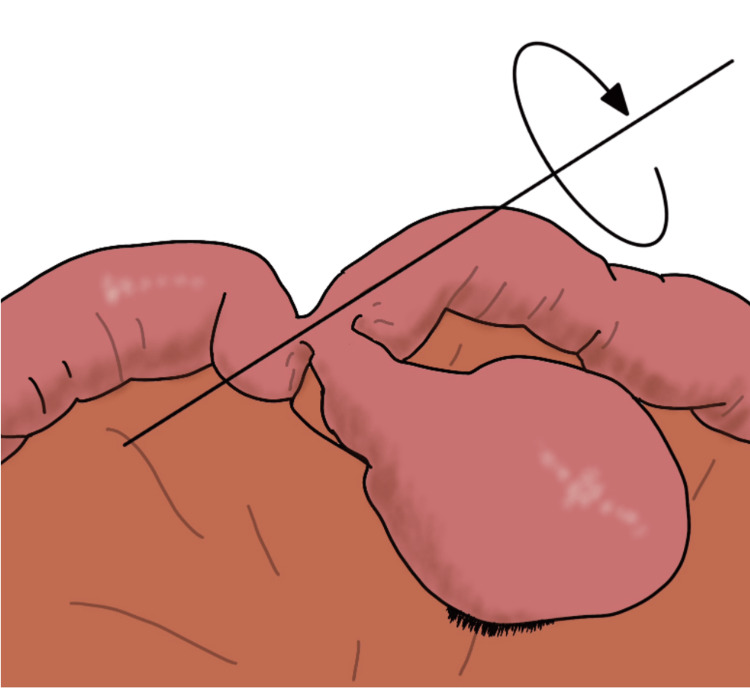
Axial torsion Image credit: Author Katsudai Shirakabe

**Figure 4:**
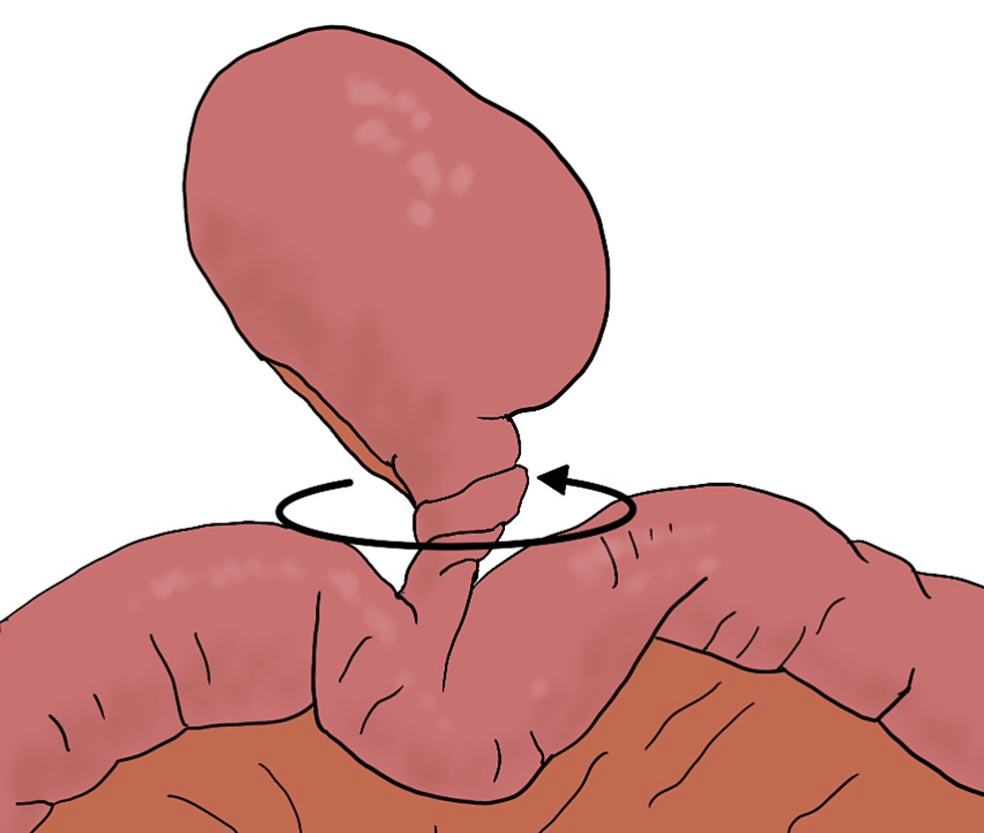
Torsion of the neck Image credit: Author Katsudai Shirakabe

The incidence of Meckel’s diverticulum in the general population is 2% [1], and only 4% of patients with Meckel's diverticulum become symptomatic [2]. The most common complication of Meckel’s diverticulum is an intestinal obstruction(36.5%) followed by intussusception(13.7%), diverticulitis(12.7%), and torsion (3.2%) [4]. There are two types of torsion: axial torsion (Figure 3), in which the ileum twists around the attachment of the diverticulum; and torsion of the neck (Figure 4), in which the diverticulum itself twists at the pedicle. The most common type of diverticular torsion is axial torsion, whereas torsion of the pedicle is extremely rare [5]. 

In this case, contrast-enhanced CT was performed. However, a definitive diagnosis was difficult to establish before surgery. The patient presented with panperitonitis, therefore, emergency surgery was performed to confirm the diagnosis.

It is often difficult to diagnose the disease using 99mTc-pertechnetate scintigraphy or small bowel contrast. In the reported case, there was no preoperative diagnosis of torsion of Meckel's diverticulum. However, the patient was operated on with the assumption of a preoperative diagnosis of strangulated bowel obstruction or acute appendicitis, making preoperative diagnosis very difficult.

The treatment of diverticular torsion of Meckel's diverticulum involves wedge resection of the diverticulum or partial small bowel resection. Laparoscopic surgery may be useful though it is extremely difficult in cases of severe small bowel dilatation [6]. In the present case, the small intestine was not decompressed, and no preoperative diagnosis was made; however, the use of an adjunctive laparoscope allowed the operation to be performed through a small incision.

When an asymptomatic Meckel's diverticulum is discovered during surgery for another disease, the decision to resect to prevent future complications remains a controversial issue. Mackey et al. [7] reported that diverticula larger than 2 cm in length, and Leijonmarck et al. [8] reported that diverticula larger than 4 cm in length are more likely to cause complications.

However, they suggested that prophylactic resection should be carefully considered. Although there are no clear criteria for prophylactic resection, it is reasonable to consider comorbidities and the general condition of patients.

## Conclusions

Common complications of Meckel's diverticulum include bowel obstruction and intussusception. However, torsion is extremely rare. As it is extremely difficult to confirm the diagnosis based on preoperative imaging alone, laparoscopic diagnosis and treatment are very useful. Hence, early laparoscopic surgery is recommended when this disease is suspected. However, if an asymptomatic Meckel's diverticulum is found during surgery for other diseases, resection should be considered based on the comorbidities and general condition of the patient.

## References

[1] Turgeon DK, Barnett JL (1990). Meckel's diverticulum. Am J Gastroenterol.

[2] Soltero MJ, Bill AH (1976). The natural history of Meckel's diverticulum and its relation to incidental removal. A study of 202 cases of diseased Meckel's Diverticulum found in King County, Washington, over a fifteen year period.. Am J Surg.

[3] Leonidas JC, Germann DR (1974). Technetium-99m pertechnetate imaging in diagnosis of Meckel's diverticulum. Arch Dis Child.

[4] Yamaguchi M, Takeuchi S, Awazu S (1978). Meckel's diverticulum. Investigation of 600 patients in Japanese literature. Am J Surg.

[5] Parab SV, Salve PG, Dahiphale A, Thakare R, Aiwale A (2017). Axial torsion of Meckel's diverticulum: a rare case report. J Clin Diagn Res.

[6] Kohga A, Yamashita K, Hasegawa Y (2017). Torsion of atypical Meckel's diverticulum treated by laparoscopic-assisted surgery. Case Rep Med.

[7] Mackey WC, Dineen P (1983). A fifty year experience with Meckel's diverticulum. Surg Gynecol Obstet.

[8] Leijonmarck CE, Bonman-Sandelin K, Frisell J, Räf L (1986). Meckel's diverticulum in the adult. Br J Surg.

